# Housing conditions change and its association with health among middle-aged and older Chinese

**DOI:** 10.1093/geroni/igaf122.3003

**Published:** 2025-12-31

**Authors:** Peiyi Lu, Tarani Chandola, Vivian Lou

**Affiliations:** The University of Hong Kong, Hong Kong, China (People’s Republic); The University of Hong Kong, Hong Kong, China (People’s Republic); The University of Hong Kong, Hong Kong, China (People’s Republic)

## Abstract

Housing, as an important and modifiable social determinant of health, is closely related to health. Improvement of housing conditions therefore presents an opportunity to promote health. However, prior research mostly focused on one single health outcome and lacked evidence from developing countries. This study investigated the relationship between housing condition changes and various health domains among middle-aged and older Chinese. Longitudinal data from China Health and Retirement Longitudinal Study were used. Participants (aged 45+, N = 8,423) reported if their homes had some amenities (e.g., barrier-free facilities, toilet) in 2011-2013. Participants’ mobility limitations, depressive symptoms, and lung function were assessed in 2013-2018. Regression models with interaction terms examined if housing condition changes modified the association between baseline housing and health. Respondents reported improvement in the physical environment and utilities from 2011 to 2013. Individuals living in homes with better physical environment (i.e., barrier-free, bathing/toileting facilities) and more utilities (i.e., gas, water, electricity, telephone and internet) reported better health (IRR< 1 for mobility limitation and depressive symptoms, b > 0 for lung function, Ps < 0.05). Additionally, home improvement in utilities lowered the risk of mobility limitations and improved lung function especially among individuals with no or few utilities at baseline. However, home improvement in physical environment did not modify the relationship between baseline physical environment and health. Our findings suggested many older Chinese resided in homes lacking essential aging-friendly amenities, which was associated with worse health. Enhancing utilities at home has the potential to modify the negative impact of earlier housing conditions on functional and lung health.

